# Identification of Three Genes Encoding for the Late Acyltransferases of Lipid A in *Cronobacter sakazakii*

**DOI:** 10.3390/md11020377

**Published:** 2013-01-31

**Authors:** Liping Cai, Yanyan Li, Guanjun Tao, Wen Guo, Chan Zhang, Xiaoyuan Wang

**Affiliations:** 1 State Key Laboratory of Food Science and Technology, Jiangnan University, 1800 Lihu Road, Wuxi 214122, China; E-Mails: cailiping7659617@sina.com (L.C.); yanyanli1123@hotmail.com (Y.L.); taogj@sina.com (G.T.); guowen0807@163.com (W.G.); zhangchan622@163.com (C.Z.); 2 Key Laboratory of Industrial Biotechnology of Ministry of Education, Jiangnan University, 1800 Lihu Road, Wuxi 214122, China

**Keywords:** * Cronobacter sakazakii*, lipid A, acyltransferase, *lpxL*, *lpxM*, *lpxP*

## Abstract

Lipid A, the hydrophobic anchor of lipopolysaccharide, is an essential component in the outer membrane of most Gram-negative bacteria. Food-borne pathogen *Cronobacter sakazakii* synthesizes two lipid A species, differing by the length of the secondary acyl chain. In this work, we identified three genes ESA02293, ESA02951 and ESA01386 encoding for the late acyltransferases of lipid A biosynthesis pathway in *C. sakazakii*. Based on the sequence alignment, proteins YP_001438378.1 encoded by ESA02293, YP_001439016.1 encoded by ESA02951, and YP_001437482.1 encoded by ESA01386 are homologous to *E. coli* LpxL, LpxP and LpxM, respectively. Functions of the three acyltransferases were confirmed by overexpressing the genes in *E. coli*, isolating lipid As and analyzing their structures using an ESI/MS. *C. sakazakii* LpxL and LpxM transfer a C14:0 secondary acyl chain to the 2′- and 3′-position of lipid A, respectively. *C. sakazakii* LpxP can transfer either a C16:1 or a C14:0 secondary acyl chains to the 2′-position of lipid A.

## 1. Introduction

Lipid Ais the hydrophobic anchor of lipopolysaccharide in the outer membrane of Gram-negative bacteria [1]. It is recognized by the TLR4/MD2 receptor of the innate immune system, which triggers an inﬂammatory response accompanied by massive over-production of cytokines and leads to Gram-negative septic shock [2,3,4]. *Cronobacter sakazakii* is a Gram-negative bacterial pathogen associated with contaminated, temperature-abused, powdered infant formula, which can cause meningitis and enterocolitis in neonates. *Cronobacter sakazakii* synthesizes two lipid A species, but the biosynthesis of lipid A in *C. sakazakii* has not been well characterized [5,6].

In *E. coli*, lipid A contains four 3-hydroxyacyl chains at the 2-, 3-, 2′-, and 3′-positions, and two acyl chains linked to the 2′-, and 3′-hydroxyacyl chains, forming acyloxyacyl moieties [7]. The four primary 3-hydroxyacyl chains are added by acyltransferases LpxA [8] and LpxD [9,10], and the two secondary acyl chains are added by late acyltransferases LpxL, LpxP and LpxM. *E. coli* LpxL transfers a C12:0 secondary acyl chain to the 2′-position of lipid A [11,12,13], LpxP has high similarity to LpxL, and can transfer a C16:0 secondary acyl chain to the 2′-position of lipid A at low temperature (12 °C) [14,15], LpxM transfers a C14:0 secondary acyl chain to the 3′-position of lipid A [16]. In contrast to *E. coli*, which synthesizes only one lipid A species, *C. sakazakii*, synthesizes two lipid A species, differing on the length of the secondary acyl chains at 2′- and 3′-positions. Late acyltransferases of lipid A in *C. sakazakii* have not been reported.

In this work, we identified three genes encoding the late acyltransferases of lipid A in *C. sakazakii* BAA894. Three enzymes encoded by genes ESA02293, ESA02952 and ESA01386 are homologous to *E. coli* LpxL, LpxP and LpxM, respectively. Like *E. coli* LpxM, *C. sakazakii* LpxM transfers a C14:0 secondary acyl chain to the 3′-position of lipid A, *C. sakazakii* LpxL and LpxP, however, functions differently from *E. coli* LpxL and LpxP. *C. sakazakii* LpxL transfers a C14:0 secondary acyl chain to the 2′-position of lipid A, while *C. sakazakii* LpxP can transfer either a C16:1 or a C14:0 secondary acyl chain to the 2′-position of lipid A even at ambient temperatures. These three acyltransferases might play important roles in biosynthesis of lipid A in *C. sakazakii*.

## 2. Results and Discussion

### 2.1. Three Genes Encoding Late Acyltransferases Were Found in *C. sakazakii* BAA894

**Figure 1:**
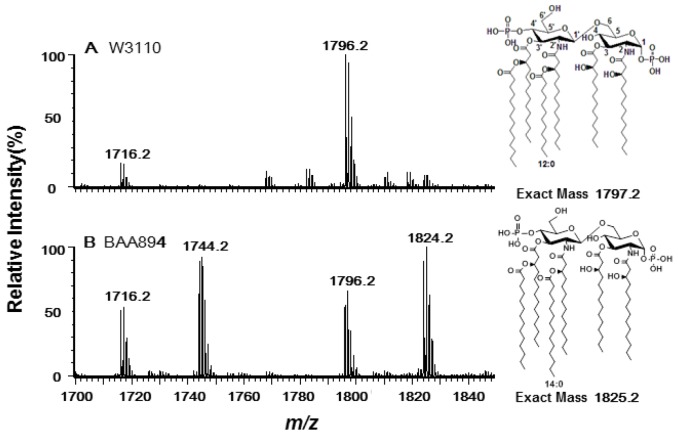
Negative ion electrospray ionization (ESI)/MS analysis of lipid A purified from *E. coli* W3110 (**A**) and *C. sakazakii* BAA894 (**B**). The chemical structures of the major lipid A species are shown on the right.

Lipid A samples were extracted from *E. coli* W3110 and *C. sakazakii* BAA894, respectively, and analyzed using ESI/MS in the negative ion mode (Figure 1). Two majorions at *m/z* 1796.1 and 1716.2 were observed in the spectrum of *E. coli* Lipid A as expected (Figure 1A). The peak at *m/z* 1796.1 was created by the molecular ion [M − H]^−^ of lipid A; the peak at *m/z* 1716.2 arisen by loss of a phosphate from the lipid A during the extraction procedure [5]. The spectrum of *C. sakazakii* lipid A showed four major peaks at *m/z* 1716.1, 1744.1, 1796.1 and 1824.1, respectively (Figure 1B). Two of the peaks (*m/z* 1716.1 and 1796.1) are the same to that of *E. coli* lipid A, while the other two peaks (*m/z* 1744.1 and 1824.1) differ from the first two by 28 Da, suggesting that *C. sakazakii* BAA894 synthesizes two different species of lipid A which differ at the length of secondary fatty acyl chains at 2′ and 3′-position [6].

**Figure 2:**
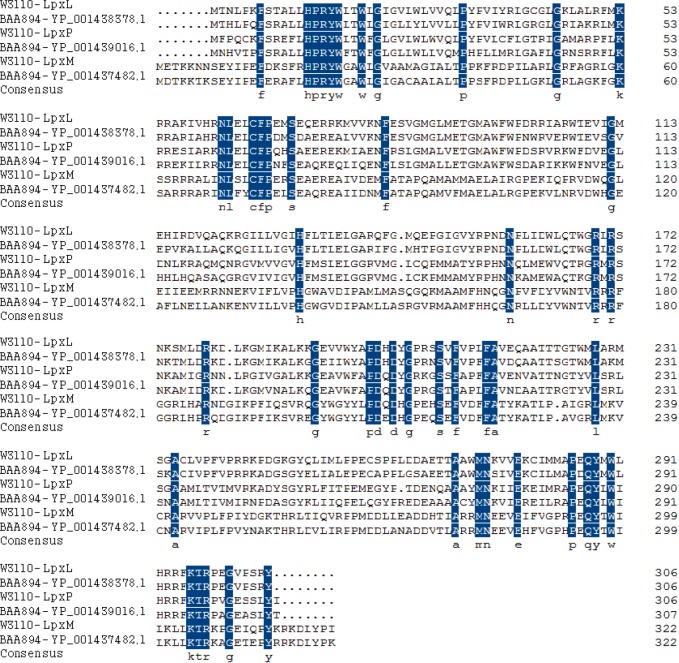
Sequence alignment of three putative late acyltransferases in *C. sakazakii* BAA894 with *E. coli* LpxL, LpxM, and LpxP. The consensus residues are emphasized.

To explore the nature of diversity of the secondary fatty acyl chains of lipid A in *C. sakazakii*, BLASTp search against the genome of *C. sakazakii* BAA894 was performed, using *E. coli* LpxL, LpxP and LpxM as queries. Three late acyltransferases YP_001438378.1, YP_001439016.1, and YP_001437482.1 were revealed and the sequence alignments of all these proteins are shown in Figure 2. YP_001438378.1 is encoded by ESA02293, contains 306 amino acids, and shares 79%, 51% and 31% identity to *E. coli* LpxL, LpxP and LpxM, respectively. YP_001439016.1 is encoded by ESA02951, contains 307 amino acids, and shares 55%, 71% and 28% identity to *E. coli* LpxL, LpxP and LpxM, respectively. YP_001437482.1 is encoded by ESA01386, contains 322 amino acids, and shares 30%, 28% and 75% identity to *E. coli* LpxL, LpxP and LpxM, respectively. Based on the sequence similarity, YP_001438378.1 is proposed as *C. sakazakii* LpxL, YP_001439016.1 as *C. sakazakii* LpxP and YP_001437482.1 as *C. sakazakii* LpxM.

To confirm the function of the three putative acyltransferases, genes ESA02293, ESA02951 and ESA01386 were cloned into pWSK29, and transformed into *E. coli* mutant MLK1067 and MKV15b. MLK1067 was constructed by inactivating *lpxM* in *E. coli* and can only synthesize pentaacylated lipid A [16]; MKV15b is a Lpxl, LpxM, LpxP triple *E. coli* mutant and could only synthesize tetraacylated lipid A [17].

### 2.2. Acyltransferases Encoded by ESA02293 and ESA02952 Are Homologous to *E. coli* LpxL and LpxP, Respectively

**Figure 3:**
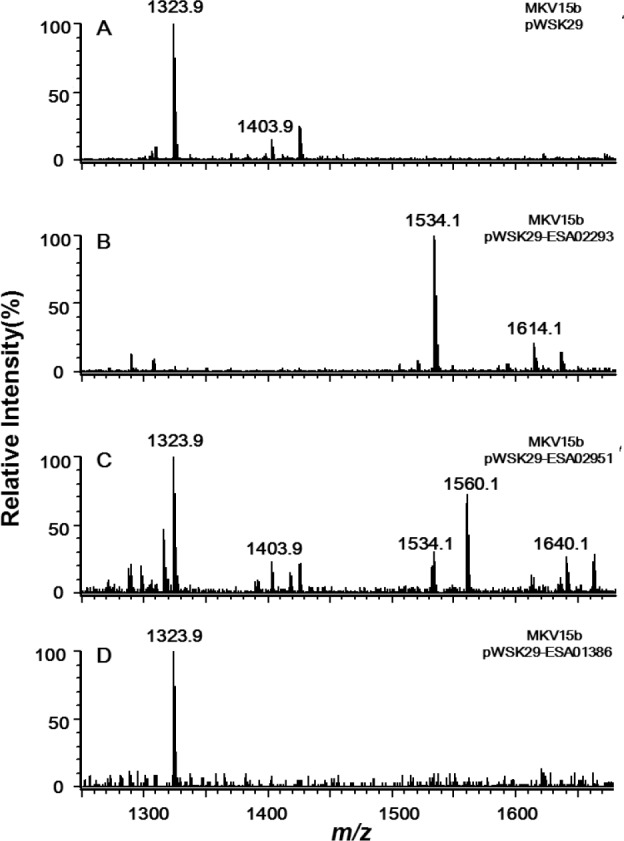
Negative ion ESI/MS analysis of lipid A isolated from *E. coli* MKV15b/pWSK29 (**A**), MKV15b/pWSK29-ESA02293 (**B**), MKV15b/pWSK29-ESA02951 (**C**) and MKV15b/pWSK29-ESA01386 (**D**).

Lipid A and its derivatives were isolated from MKV15b/PWSK29, MK15b/pWSK29-ESA02293, MK15b/pWSK29-ESA02951, and MK15b/pWSK29-ESA01386, respectively, and analyzed by ESI/MS (Figure 3).

Two major ions at *m/z* 1403.9 and 1323.9 were observed in the spectrum of lipid A from the control MKV15b/pWSK29 (Figure 3A). The peak at *m/z* 1403.9 is created by the molecular ion [M − H]^−^ of lipid A containing two phosphate groups and four primary fatty acid chains; the peak at *m/z* 1323.9 arises by loss of a phosphate from the molecular ion during the extraction procedure. In the spectrum of lipid A sample from MKV15b/pWSK29-ESA02293, two major ions were also observed (Figure 3B). The molecular ion at *m/z* 1614.1 differs from that of the lipid A from MKV15b/pWSK29 by 210 amu, that is, a C14:0 fatty acid unit. The peak at *m/z* 1534.1 arises by loss of a phosphate from the molecular ion. The results indicate that the enzyme encoded by ESA02293 transfers a C14:0 secondary acyl chain to the 2′-position of lipid A. 

Six peaks were observed in the spectrum of lipid A sample from MKV15b/pWSK29-ESA02951 (Figure 3C). Peaks at *m/z* 1323.9 and 1403.9 are the same to that found in spectrum of lipid A from both MKV15b/pWSK29. Peaks at *m/z* 1534.1 and 1614.1 are the same to that found in spectrum of lipid A from MKV15b/pWSK29-ESA02293. This indicates that the enzyme encoded by ESA02951 can transfer a C14:0 secondary acyl chain to the 2′-position of lipid A, but its efficiency is not as high as the enzyme encoded by ESA02293. In addition, there were two new peaks at *m/z* 1560.1 and 1640.1 in the spectrum of lipid A from MKV15b/pWSK29-ESA02951; the former might arise by loss of a phosphate form the later. The molecular ion at *m/z* 1640.1 differs from that of the molecular ion of lipid A from MKV15b/pWSK29 by 236 amu moiety, that is, a C16:1 fatty acid unit. The results indicate that the enzyme encoded by ESA02951 could transfer a C14:0 or a C16:1 secondary acyl chain to the 2′-position of lipid A.

The spectrum of lipid A sample from MK15b/pWSK29-ESA01386 only showed a peak at *m/z* 1323.9 created by the molecular ion losing a phosphate, which is exactly the same as that of the control strain MKV15b/pWSK29 (Figure 3D), suggesting the enzyme encoded by the gene ESA01386 does not function on the substrate lipid A with only four primary fatty acyl chains. Based on the analysis of sequence and function, the acyltransferase encoded by ESA02293 should be the LpxL in *C. sakazakii*, and the acyltransferase encoded by ESA_02952 should be the LpxP in *C. sakazakii*.

Because there is a second molecular ion at *m/z* 1796.2 in the *C. sakazakii* lipid A mass spectrum (Figure 1B), there might be another acyltransferase in *C. sakazakii* which transfers a C12:0 secondary acyl chain to lipid A. This acyltransferase may have no sequence similarity to LpxL. This situation was observed for LpxH, a key enzyme in the lipid A biosynthesis of *E. coli*. A new enzyme LpxI which has no sequence similarity to LpxH but generates the same products by a different route was recently found in *Caulobacter crescentus* [18]. Unlike *E. coli* LpxP which transfers a C16:1 acyl chain only at low temperature (12 °C), *C. sakazakii* LpxP can function at ambient temperature and can transfer either a C14:0 or a C16:1 secondary acyl chains. It would be interesting to study the structural difference of these two LpxP proteins to figure out the machnism for their different substrate specificity and temperature regulation.

### 2.3. Acyltransferase Encoded by ESA01386 Is the Homologue of *E. coli* LpxM

**Figure 4:**
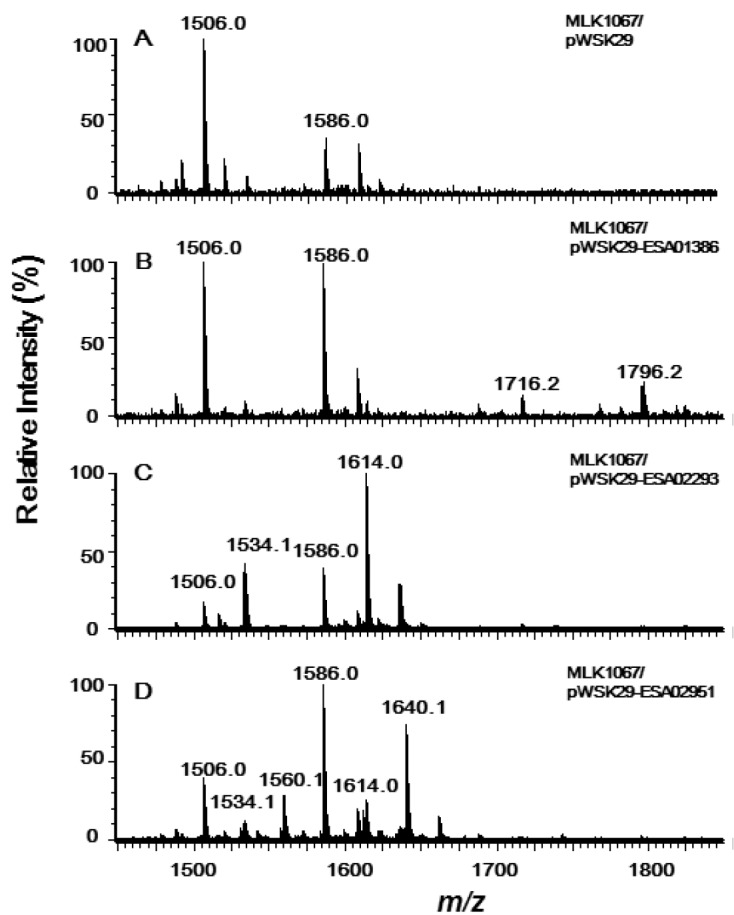
Negative ion ESI/MS analysis of lipid A isolated from *E. coli* MLK1067/pWSK29 (**A**), MLK1067/pWSK29-ESA01386 (**B**), MLK1067/pWSK29-ESA02293 (**C**) and MLK1067/pWSK29-ESA02951 (**D**).

Lipid A and its derivatives were isolated from MLK1067/PWSK29, MLK1067/pWSK29-ESA02293, MLK1067/pWSK29-ESA02951, and MLK1067/pWSK29-ESA01386, respectively, and analyzed by ESI/MS (Figure 4). 

Lipid A isolated from MLK1067/pWSK29 showed two major peaks in the spectrum; one is the molecular ion [M − H]^−^ at *m/z* 1586.0 [6], and the other at *m/z* 1506.0 is derived by loss of a phosphate form the molecular ion (Figure 4A). Two additional peaks at *m/z* 1716.2 and 1796.2 were observed in the spectrum of lipid A samples from MLK1067/pWSK29-ESA01386 (Figure 4B). The peak at *m/z* 1796.2 should be from the molecular ion [M − H]^−^, and the peak at *m/z* 1716.2 is derived from the molecular ion by loss of a phosphate. The *m/z* value difference between the two molecular ions of lipid A from MLK1067/pWSK29 and MLK1067/pWSK29-ESA01386 is 210 amu, that is, a C14:0 acyl chain. This suggests that the enzyme encoded by ESA01386 could transfer a C14:0 fatty acids to the 3′-position of lipid A, but its efficiency is not as high as *E. coli* LpxM because the majority of lipid A in the cell are in the pentaacylated form. Considering *C. sakazakii* LpxL transfers a C14:0 secondary acyl chain to the 2′-position of lipid A (Figure 3B), the pentaacylated lipid A with a C12:0 secondary fatty acyl chain in *E. coli* MLK1067 might not the ideal substrate for this enzyme. Based on the analysis of sequence and function, the acyltransferase encoded by ESA01386 should be the LpxM in *C. sakazakii*. 

The peaks at *m/z* 1796.2 and 1716.2 were not observed in the spectra of lipid A samples from MLK1067/pWSK29-ESA02293 and MLK1067/pWSK29-ESA02951, confirming that the enzymes encoded by ESA02293 and ESA02951 cannot add any acyl chains to the 3′-position of lipid A. In addition to the two peaks at *m/z* 1506.0 and 1586.0 observed in the control, two addition peaks at *m/z* 1534.1 and 1614.0 were observed in the spectrum of lipid A from MLK1067/pWSK29-ESA02293 (Figure 4C), confirming again that *C. sakazakii* LpxL prefers to transfer a C14:0 acyl chain to the 2′-position of lipid A. 

Six peaks were observed in the spectrum of lipid A sample from MLK1067/pWSK29-ESA02951 (Figure 4D). Peaks at *m/z* 1506.0 and 1586.0 are the same to that found in spectrum of lipid A from MLK1067/pWSK29. Peaks at *m/z* 1534.1 and 1614.1 are the same to that found in spectrum of lipid A from MKV15b/pWSK29-ESA02293 or MLK1067/pWSK29-ESA02293. Peaks at *m/z* 1560.1 and 1640.1 are the same to that found in the spectrum of lipid A from MKV15b/pWSK29-ESA02951 (Figure 3C). These data further confirm that *C. sakazakii* LpxP can transfer either a C16:1 or a C14:0 secondary acyl chain to the 2′-position of lipid A.

## 3. Experimental Section

### 3.1. DNA Preparation and PCR Techniques

Plasmid DNA was prepared by using the EZ-10 spin column plasmid mini-preps kit from Bio Basic Inc. (Markham, Canada). Fifty microliters of PCR reaction mixture was used in this study, which contains 5 μL 10× Extaq buffer, 4 μL dNTP (2.5 mM each), 1 μL plasmid template, 1 μL forward primer (20 μM), 1 μL reverse primer (20 μM), and 0.5 μL Extaq polymerase. The PCR reaction was started at 94 °C for 5 min, followed by 35 cycles of denaturation (30 s at 94 °C), annealing, and extension. PCR products were puriﬁed using the TIAN gel puriﬁcation kit from Tiangen (Beijing, China). Primers synthesis and DNA sequencing were performed by Sangon (Shanghai, China).

### 3.2. Construction of Plasmids Containing Genes ESA02293, ESA02951 or ESA01386

ESA02293, ESA02951, and ESA01386 genes were obtained by PCR, using the genome of *C. sakazakii* BAA-894 as a template. All the primers used for the clones were listed in Table 1. Briefly, all the forward primers contained an XbaI site, and all the reverse primers contained a BamHI site. The PCR products were purified, digested with XbaI and BamHI, and ligated into the vector pWSK29 which was similarly digested. The resulting plasmids were designated as pWSK29-ESA02293, pWSK29-ESA02951 and pWSK29-ESA01386, respectively. These plasmids were transformed into *E. coli* mutants MLK1067 [5] and MKV15b [17], resulting strains MLK1067/pWSK29-ESA02293, MLK1067/pWSK29-ESA02951, MLK1067/pWSK29-ESA01386, MLV15b/pWSK29-ESA02293, MLV15b/pWSK29-ESA02951, and MLV15b/pWSK29-ESA01386. All strains used in this study are listed in Table 1. All *C. sakazakii* and *E. coli* stains were grown in LB media at 37 °C. 100 μg/mL ampicillin, 50 μg/mL kanamycin, or 20 μg/mL chloramphenicol were used when necessary.

**Table 1:** Stains and primers used in this study.

Strains or oligonucleotides	Relevant characteristic or sequence	Source or purpose
**Strains**		
ATCC BAA894	Wild-type *C. sakazakii*	ATCC
W3110	Wide-type *E. coli*	ATCC
MKV15b	*W3110 lpxL* *::Tn10 lpxM* *::Ωcam lpxP* *::kan*	[17]
MKV15b/pWSK29	MKV15b harboring pWSK29	This work
MKV15b/pWSK29-ESA02293	MKV15b harboring pWSK29-ESA02293	This work
MKV15b/pWSK29-ESA02951	MKV15b harboring pWSK29-ESA02951	This work
MKV15b/pWSK29-ESA01386	MKV15b harboring pWSK29-ESA01386	This work
MLK1067	*W3110 lpxM* *::Ωcam*	[16]
MLK1067/pWSK29	MLK1067 harboring pWSK29	This work
MLK1067/pWSK29-ESA01386	MLK1067 harboring pWSK29-ESA01386	This work
MLK1067/pWSK29-ESA02293	MLK1067 harboring pWSK29-ESA02293	This work
MLK1067/pWSK29-ESA02951	MLK1067 harboring pWSK29-ESA02951	This work
**Oligonucleotides**		
F-ESA02293	GCTCTAGAATGACGCATTTACCGCAATT	Forward primer for cloning ESA-02293
R-ESA02293	CGGGATCCTTAGTAGCGGGACGGCACGC	Reverse primer for cloning ESA-02293
F-ESA02951	GCTCTAGAATGAATCACGTCACGCCTTTTTC	Forward primer for cloning ESA-02951
R-ESA02951	CGGGATCCTCAGGTATAGAGCGACGCTTC	Reverse primer for cloning ESA-02951
F-ESA01386	GCTCTAGAATGGACACAAAAAAAACAAAAAGTG	Forward primer for cloning ESA-01386
R-ESA01386	CGGGATCCTTATTTCGGATAAAGATCTTTGC	Reverse primer for cloning ESA-01386

### 3.3. Lipid A Isolation from Different *E. coli* Strains

Lipid A was isolated using the Bligh-Dyer method [19]. Briefly, 200 mL cultures were grown in LB broth at 37 °C to OD_600_ of 1.0. Cells were harvested by centrifugation at 8000 rpm and washed twice with ddH_2_O. The cell pellet was resuspended in 76 mL of a single-phase Bligh-Dyer mixture containing chloroform/methanol/H_2_O (1:2:0.8, v/v/v), incubated with stirring at room temperature for 1 h. The insoluble debris was collected by centrifugation at 2000 rpm for 20 min and washed three times with single-phase Bligh-Dyer mixture. The debris was resuspended in 27 mL of 12.5 mM sodium acetate (pH 4.5), heated at 100 °C for 30 min, and cooled to room temperature. The suspension was mixed with 30 mL chloroform and 30 mL methanol, and the two phases were separated by centrifugation. The lower phase contains lipid A, and was dried under a stream of nitrogen.

### 3.4. Mass Spectrometry Analysis

All the mass spectra of lipid A samples were acquired on a Waters SYNAPT quadrupole time-of-flight mass spectrometer equipped with an electrospray ionization (ESI) source. Lipid A samples were dissolved in chloroform/methanol (2:1, v/v) and subjected to ESI/MS in the negative ion mode. Data acquisition and analysis were performed using Mass Lynx V4.1 software [20].

## 4. Conclusions

Unlike *E. coli* which usually synthesizes only one lipid A species, *C. sakazakii* BAA894 synthesizes two different species of lipid A, differing on the length of the secondary acyl chain at 2′-position. Here, we identified three late acyltransferases in *C. sakazakii* BAA894. The enzyme encoded by the gene ESA02293 is homologous to *E. coli* LpxL, and transfers a C14:0 acyl chain to the 2′-position of lipid A. The enzyme encoded by the gene ESA_02953 is homologous to *E. coli* LpxP, and transfers either a C16:1 or a C14:0 acyl chains to the 2′-position of lipid A. The enzyme encoded by ESA01386 is homologous to *E. coli* LpxM, and transfer a C14:0 acyl chain to the lipid A at 3′-position. Since the number and length of secondary acyl chains of lipid A are important for the recognition of TLR4/MD2 to LPS, these late acyltransferases found in this study might be useful to construct various lipid A moieties for studying the immune responses.
